# The Great Mimicker: Pulmonary Embolism Presenting as Flank Pain in a Sickle Cell Disease Patient

**DOI:** 10.7759/cureus.39924

**Published:** 2023-06-03

**Authors:** Maryam A AL-Ramadhan, Amena A AL-Janobi

**Affiliations:** 1 Internal Medicine, Qatif Central Hospital, Qatif, SAU; 2 Internal and Pulmonary Medicine, Qatif Central Hospital, Qatif, SAU

**Keywords:** pulmonary infarction, abdominal pain, flank pain, sickle cell disease (scd), pulmonary embolism

## Abstract

Pulmonary embolism can be a challenging condition for physicians to manage. They often have to diagnose this disease with a high fatality rate via the presence of non-specific symptoms. Another unusual presentation is abdominal pain, which can delay diagnosis due to a broad differential.

We report the case of a 30-year-old female with a history of sickle cell anemia who presented to the Emergency Department with several days of right flank pain and urinary symptoms. Unfortunately, her initial urine analysis and chest radiograph could have been misdiagnosed as pyelonephritis. Early diagnosis and timely treatment are critical factors in reducing the mortality rate from pulmonary embolism.

## Introduction

Pulmonary embolism (PE) can be challenging for physicians, from its diagnosis with non-specific symptoms that the patient may present with, for example, pleuritic chest pain, dyspnea, cough, or pleural effusion, to its outcomes with a high fatality rate [1]. Therefore, even sudden death can be considered a presentation of PE, and the diagnosis is made after an autopsy. Another unusual presentation is abdominal pain, which can delay diagnosis due to a wide differential [2]. Here, we present a sickle cell disease female who complained of abdominal pain and was found to have a pulmonary embolism.

## Case presentation

A 30-year-old female presented to the Emergency Department with a complaint of right flank pain for five days; it was dull and radiated to the right upper quadrant of the abdomen (RUQ). She also had dysuria and difficult micturition, low-grade fever, and vomiting. Her medical history was significant for sickle cell disease (SCD). Surgical history was remarkable for cholecystectomy and splenectomy.

Her initial vital signs showed a temperature of 38.1 °C, heart rate of 97 beats per minute, blood pressure of 125/74 mm Hg, respiratory rate of 19 breaths/min, and oxygen saturation of 99% on room air. Physical examination showed mild right costovertebral angle and RUQ tenderness with no other systemic signs.

Laboratory results revealed hemoglobin (Hgb) 10 mg/dl, WBC 12.8, and platelet (PLT) 766; liver and renal function were normal, including the pancreatic enzyme. Urine examination showed leucocytosis with one positive leucocyte esterase. Culture results were pending. Chest X-ray was clear. She was admitted with a urinary tract infection (UTI) and suspected of pyelonephritis, started on ceftriaxone 2-gram intravenous once and analgesia.

On days one through four, she continued to have RUQ pain despite antibiotics and analgesia with no other symptoms. She was afebrile with normal oxygen saturation. Renal and pelvic ultrasounds were normal, with no sign of perinephric abscess or collection. As her abdominal pain persists, we consider acute abdomen vs. abdominal crisis as she is SCD; the surgical team was involved and planned for computerized tomography (CT) scan.

On day five, the abdominal pain continued to be more severe; she developed a new dry cough with a drop in oxygen saturation to 92% on room air and a pulse of 99 beats per minute. Her chest examination revealed right side crepitation and her abdominal exam showed severe localized right upper quadrant tenderness. By this time, we had received urine and blood culture results which were negative. However, a repeated chest radiograph showed new infiltration in the right lower lung zone and pleural effusion.

**Figure 1:**
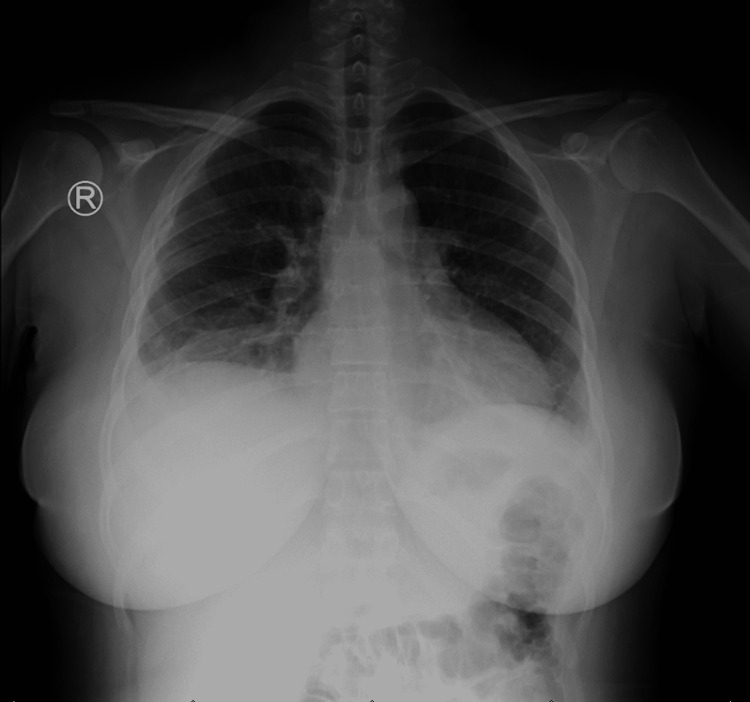
Chest x-ray posteroanterior (PA) view shows a hazy left costophrenic angle with sub-pulmonic effusions on the right side.

Hospital-acquired pneumonia (HAP)/acute chest syndrome as she is SCD (Figure 1) was raised with this clinical deterioration, although it was not explained by her severe abdominal pain; the antibiotic was upgraded to piperacillin-tazobactam to cover HAP.

**Figure 2:**
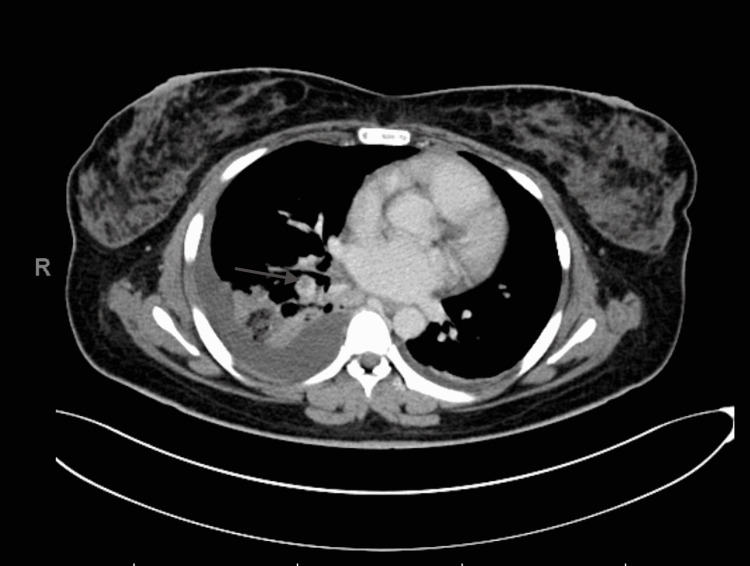
Transverse CT chest view shows a filling defect in the sub-segmental branches of the pulmonary arteries on the right side representing pulmonary embolism associated with consolidation and bilateral pleural effusion (more over the right side)

On day six, CT with contrast was done. Surprisingly, it showed a right-sided subsegmental pulmonary embolism with pulmonary infarction (Figure 2), minimal free fluid at the porta hepatis, and no abscess or other fluid collection in the abdomen.

She was started on therapeutic anticoagulation. Within a day of anticoagulation, her abdominal pain improved significantly with no other symptoms; later, she was discharged on oral apixaban. During outpatient follow-up, she was asymptomatic; repeated CXR shows resolution.

## Discussion

Abdominal pain as a primary complaint often points the physician to a broad differential diagnosis. Usually, they will not consider pulmonary embolism as a cause, especially if the patient does not have other classical signs or symptoms such as chest pain, dyspnea, tachypnea, or tachycardia [3].

This initial patient presentation pointed toward pyelonephritis with flank pain, fever, and urine exam suggestive of urinary tract infection. However, other investigations, such as the renal US, did not explain her persistent abdominal pain. For this reason, the patient needed further diagnostic testing as abdominal CT, which showed subsegmental branches of pulmonary embolism. 

Current literature has hypothesized that abdominal pain is due to right heart strain, which results in congestion of the liver, Glisson’s capsule distension, and bowel congestion. Furthermore, pulmonary infarction causes diaphragmatic pleurisy and tension on the nerve endings in the parietal pleura [4].

As a pathogenesis for thrombosis, Virchow described a triad in 1846: venous stasis, hypercoagulation states, and inflammation of the vascular endothelium. If there is a defect in any one of these components, then a thrombus is more likely to form [5]. Sickle cell disease is one of the risk factors for thrombosis [6]. As in our patient, pulmonary embolism should be in the differential diagnosis, but urinary symptoms and urine analysis clouded her initial diagnosis at the time of presentation. Due to the delay in her improvement, further investigation was done, and it showed a pulmonary embolism.

## Conclusions

Abdominal pain is one of the unusual symptoms of a pulmonary embolism that patients can experience. Making physicians aware of this presentation may help them diagnose and treat the condition much earlier.
